# A dynamic Dab2 keeps myosin VI stably on track

**DOI:** 10.1016/j.jbc.2021.100640

**Published:** 2021-05-03

**Authors:** Joseph A. Cirilo, Christopher M. Yengo

**Affiliations:** Department of Cellular and Molecular Physiology, Penn State College of Medicine, Hershey, Pennsylvania, USA

**Keywords:** myosin, actin, cytoskeleton, adaptor protein, clathrin, endocytosis, DAB2, DNA origami, FRET, single molecule

## Abstract

Myosins are actin-based motor proteins known to perform a variety of different mechanical tasks in cells including transporting cargo, generating tension, and linking the cytoskeleton and membrane. Myosins that function as transporters often form complexes with adaptor proteins and vesicular membranes, making it unclear how they transport their cargo through the actin cytoskeletal network. Rai *et al.* now use single-molecule kinetics, FRET, and DNA origami scaffolds that mimic motor–adaptor complexes to reveal that the myosin VI-Dab2 complex, which is held together weakly and turns over rapidly, can facilitate processive transport without disruption of the cytoskeleton.

Many cytoskeletal motor proteins, including members of the myosin, kinesin, and dynein superfamilies, are involved in the directed transport of cargo to specific subcellular compartments. The adaptor proteins that link these motors to their respective cargo are now recognized as key players in regulating transport. For example, the myosin motor MYO5A forms complexes with the adaptor proteins melanophilin and RAB27A to transport pigment-containing melanosomes in melanophores (1). Mutations in either of these adaptor proteins can lead to disease phenotypes such as Griscelli’s syndrome, a genetic disorder characterized by pigment dilution, neurological defects, and immunodeficiency. The kinesin motor KIF5C requires interaction with its adaptors HAP1 and GRIP1 to become activated from an autoinhibited state (2). Dynein has been associated with a plethora of adaptor proteins, including Hook1, HAP1, TRAK1/2, and BICD/BICD2, several of which are associated with neurodegenerative diseases (3). These examples demonstrate some of the diversity of possible mechanisms, as well as the importance of investigating motor–adaptor interactions for understanding intercellular transport mechanisms. A study from Rai *et al.* (4) expands our understanding further, demonstrating how a dynamic adaptor protein helps its motor protein partner navigate the complex cytoskeletal network.

Myosins often transport cargo in the cell cortex, near the plasma membrane, where the actin cytoskeleton is highly organized and densely packed (5). For example, myosin VI is an unconventional myosin that plays a critical role in endocytosis, requiring its presence in the cortex. It relies on the adaptor protein Dab2, which facilitates myosin VI dimerization and links it to clathrin-coated endosomes assisted by the clathrin adaptor AP-2 (6, 7). The formation of myosin VI dimers is essential for the motor’s processivity, *i.e.*, the ability of myosin to undergo multiple catalytic cycles before disengaging with actin. Surprisingly, the Dab2–myosin VI interaction is relatively weak (6), raising the question of how this association could promote the efficient transport of clathrin-coated vesicles. In addition, previous biophysical studies found that force generated by dimeric myosins can alter the organization of the actin cytoskeleton (8), raising questions as to how myosin-based transport in the cortical actin network can reliably occur without irreparably altering actin organization.

These questions prompted Rai *et al.* (4) to explore the Dab2–myosin VI complex in more detail. The authors first utilized a FRET approach to confirm that Dab2 binds weakly to actin and dimerizes myosin VI. Single-molecule kinetics similarly confirmed that the timescale of this interaction was on the same order as the myosin VI ATPase cycle (1 s^−1^) (9). The authors then examined the processive movement of the Dab2–myosin VI complexes using single-molecule motility assays and a surface actin gliding assay, finding that the complex could efficiently generate continuous movements that were similar to artificially dimerized myosin VI controls and that single complexes of Dab2–myosin VI were capable of generating actin filament gliding. Next, the authors used DNA origami, a technique used to transform DNA into various nanostructures, to create a cargo mimic. The DNA nanostructures with Dab2–myosin VI attached were able to move long distances along a single actin filament, though not as efficiently as constitutively dimerized myosin VI. The authors then examined myosin motility on cellular actin networks that were detergent-extracted from corneal fibroblasts. In this system, Dab2–myosin VI-mediated transport of DNA nanostructures was also processive, albeit with more frequent pauses than controls. These data thus confirm that Dab2–myosin VI is capable of facilitating processive transport despite its weak and dynamic interaction.

Rai *et al.* next examined how this unusual interaction impacts the actin cytoskeleton. To accomplish this, they generated a lipid bilayer surface and decorated it with polymerized actin. Upon addition of constitutively dimerized myosin VI or myosin V, another myosin motor capable of processive movement, they observed a dramatic reorganization of the actin cytoskeleton, indicated by the formation of discrete actin foci. Interestingly, the Dab2–myosin VI complexes produced significantly fewer actin foci, while monomeric myosin VI did not cause foci. The authors went on to further examine the actin reorganizing behavior and found that it requires ATP and does not occur in the presence of a nonhydrolysable ATP analog or without nucleotide.

The results from Rai *et al.* (4) give us a new way of thinking about motor–cargo complexes in that they may be more dynamic than initially realized. This may be important to prevent rearrangement of cytoskeletal tracks, likely caused by centripetal accumulation of actomyosin in the actin foci. Other motor systems may also need to acquire methods of preserving the cytoskeleton they walk on. Indeed, processive movement of kinesin and dynein was found to cause removal of tubulin dimers in the microtubule lattice, and a self-repair process was required to compensate and prevent disassembly (10). In other situations, motors are crucial for rearranging the cytoskeleton to form cellular structures, such as formation of the mitotic spindle or cellular protrusions. Thus, the multimerization of molecular motors is important not only for their regulation and linkage to cargo but also for mediating the precise architecture of the cytoskeleton.

A number of new questions and future directions arise from the Rai *et al.* study (4). First, it will be important to examine the myosin-induced disruption of the cortical actin cytoskeletal organization in live cells, both to dissect the requirements for minimizing motor-induced cytoskeletal rearrangements inside cells and to define upstream regulators. Second, the study calls into question whether other myosin motors utilize adapter proteins to mediate their monomer/dimer equilibrium and how this contributes to their cellular function. Furthermore, this study showed that the interaction between myosin VI and Dab2 is low affinity. Other myosin–adaptor interactions, such as the interaction between MYO5A and the adaptor Slac-2a, display variable affinity throughout development (1). Taken together, this suggests that the affinity of myosin–adaptor interactions is fine-tuned for their physiological function. Perhaps studying motors in the more native environment (*e.g.*, complexed with adapter proteins and interacting with their cargo) will reveal new clues about how they are regulated and how they navigate complex cytoskeletal networks. Overall, investigating the interplay between motor multimerization and cytoskeletal organization will reveal important details for how cells accomplish a variety of mechanical tasks while preserving cytoskeletal organization in each subcellular compartment.

## Conflict of interest

The authors declare that they have no conflicts of interest with the contents of this article.
